# Serum p53 Antibody Is Not Associated with p53 Immunoreactivity in Patients with Pancreatobiliary Cancers

**DOI:** 10.1155/2013/170625

**Published:** 2013-12-18

**Authors:** Junko Umeda, Takao Itoi, Atsushi Sofuni, Fumihide Itokawa, Toshio Kurihara, Takayoshi Tsuchiya, Kentaro Ishii, Shujiro Tsuji, Nobuhito Ikeuchi, Reina Tanaka, Ryosuke Tonozuka, Mitsuyoshi Honjo, Shuntaro Mukai, Toshitaka Nagao, Hisashi Oshiro, Fuminori Moriyasu

**Affiliations:** ^1^Department of Gastroenterology and Hepatology, Tokyo Medical University, Tokyo 160-0023, Japan; ^2^Department of Pathology, Tokyo Medical University, Tokyo 160-0023, Japan

## Abstract

*Background*. Recent diagnostic imaging tests contribute to improving the diagnosis of pancreatobiliary cancers. However, it is not practical to perform these tests for all patients as screening. Thus, less-invasive and simple screening tests are still required. A method to detect the IgG antibody induced in serum against the p53 protein accumulating due to p53 gene mutation, as a biomarker, was developed around 1990. *Method*. 35 patients with pancreatic cancer, 12 patients with biliary tract cancer, and 31 patients with benign pancreatobiliary diseases were entered into this study. Measurement of serum anti-p53 antibody was conducted in all patients. In addition, the rate of p53 protein overexpression was examined in those cases that could be examined pathologically. *Result*. Among all patients in the pancreatic cancer and biliary tract cancer groups, there was no patient with serum anti-p53 antibody positive value that exceeded the standard value. The rate of p53 protein overexpression was 48.0% in the patients with pancreatobiliary cancers and 0% in the benign pancreatobiliary diseases group. *Conclusion*. Serum anti-p53 antibody measurement does not contribute to the diagnosis of pancreatobiliary cancers. Instead, traditional p53 immunostaining still appears to be valuable in combination with standard procedures.

## 1. Introduction

Recent diagnostic imaging tests such as computed tomography (CT) and magnetic resonance imaging (MRI) contribute to improving the diagnosis of pancreatobiliary cancers [1–4]. However, from the point of cost benefit, it is not practical to perform these tests for all patients as screening. Thus, less-invasive and simple screening tests are still required.

As screening tests, hematology tests are known to be the most simple and minimally invasive. CA19-9 and CEA have been reported as comparatively useful tumor markers for pancreatobiliary cancers [5–7]. On the other hand, various forms of gene mutation are now present in the cancer chemotherapy process in pancreatobiliary cancers. Among these genes, mutation of the p53 gene has been reported in various tumors [8], and even in pancreatobiliary cancers it has been found at the rate of 30~50% [9–13], and in pancreatic cancer at the rate of 60% [14]. Infiltrative cancers in particular have been reported to have a high rate of at least 70% of gene mutation [15]. Apart from the detection of such gene mutation, a method to detect the IgG antibody (serum anti-p53 antibody) induced in serum against the p53 protein accumulating due to p53 gene mutation, as a biomarker, was developed around 1990 [16]. Shimada et al. reported the usefulness of the measurement of serum anti-p53 antibody in various malignant tumors [16–20] and suggested that it is clinically useful particularly in cancers that are in the early stage comparatively, where the positive rate is high. As a novel tumor marker for esophageal cancer, colon cancer, and breast cancer, it became eligible for health insurance coverage from 2007 in Japan. Moreover, it has been shown to be useful even in the prediction of prognosis during the treatment process of cancers and relapse in postsurgery cases [21]. On other hand, it is expected to be useful in the measurement of serum anti-p53 antibody as in gastrointestinal cancer even in malignant pancreatobiliary tumors.

In the present study, serum anti-p53 antibody was measured in patients with pancreatobiliary cancer. Serum anti-p53 antibody measurement was performed for cases of pancreatobiliary cancer experienced at our institution. In addition, the rate of p53 protein overexpression was examined in those cases that could be examined pathologically.

## 2. Materials and Methods

### 2.1. Samples

35 patients with pancreatic cancer, 12 patients with biliary tract cancer (Table 1), and a control group consisting of 31 patients with benign pancreatobiliary diseases (13 with common bile duct stones, 6 with chronic pancreatitis, 4 with benign bile duct stricture, 2 with pancreatic pseudocyst, 2 with autoimmune pancreatitis, 2 with adenomyomatosis of the gallbladder, 1 with pancreatobiliary maljunction, and 1 with benign pancreatic duct stricture) (Table 2) that had been hospitalized for treatment at this institution between December 2010 and April 2011 were entered into this study. Mean age was 69.3 years (range, 51–83 years) for pancreatic cancer, 72.3 years (range, 55–88 years) for biliary tract cancer, and 65.0 years (range, 35–86 years) for benign pancreatobiliary tract disease. The male-to-female ratio was 19 : 16 for pancreatic cancer, 8 : 4 for biliary tract cancer, and 25 : 6 for benign pancreatobiliary tract disease.

### 2.2. Serum Level of CA19-9, CEA, and Anti-p53 Antibody

Serum CA19-9, CEA, and anti-p53 antibody were determined for all patients prior to treatment. The required serum sample of 0.3 mL was used to measure anti-p53 antibody by ELISA and the measurement kit was MESACUP anti-p53 TEST (Medical Biological Research Institute Inc., Tokyo, Japan). A value ≥1.30 U/mL was judged as anti-p53 positive.

### 2.3. p53 Immunohistochemical Analysis

**Figure 1:**
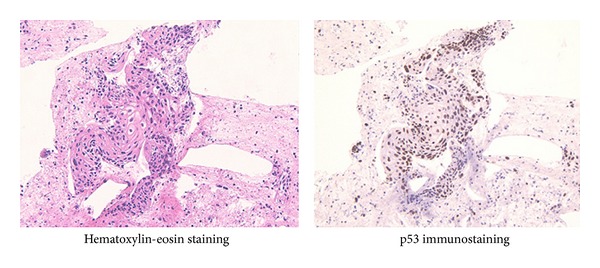
EUS-FNA specimen of pancreatic cancer with p53 protein overexpression.

In addition, immunostaining was performed with p53 protein (D0-7, DAKO, Glostrup, Denmark) by the SAB method on formalin-fixed paraffin-embedded fragments obtained from those patients from whom adequate tissue samples could be obtained by biopsy or surgical resection. Cells with nuclei stained brown were judged as positive cells. A comparison was made with HE staining (Figure 1) and the percentage of cancer cells that were positive was calculated. A rate of ≥70% was judged as overexpression of p53 protein. Moreover, a comparison was made with the staining results of noncancerous epithelium of the same fragment to determine the diagnostic performance of cancer by p53 protein overexpression.

## 3. Statistical Analysis

Diagnostic accuracy, sensitivity, and specificity of each marker were calculated and compared with the final diagnosis. Statistical analysis was performed using chi-square test. A *P* value less than 0.05 was regarded as indicating a statistically significant difference. Statistical analyses were performed with StatMate III (ATMS Co. Ltd., Tokyo, Japan).

## 4. Results

### 4.1. Serum Level of CEA and CA19-9

Mean CEA (standard value: <5.0 ng/mL) in the pancreatic cancer group was 38.33 ng/mL, the positive rate was 42.8%, mean CA19-9 (standard value: <37 U/L) was 3142.92 U/L, and the positive rate was 85.7%. In addition, mean CEA in the biliary tract cancer group was 21.42 ng/mL, the positive rate was 41.6%, mean CA19-9 was 5859.88 U/L, and the positive rate was 91.6%. In the pancreatobiliary cancer group, mean CEA was 34.01 ng/mL, the positive rate was 74.0%, mean CA19-9 was 3836.6 U/L, and the positive rate was 87.2% (Tables 3, 4, 5, and 6).

### 4.2. Serum Level of Anti-p53 Antibody

Among all patients in the pancreatic cancer and biliary tract cancer groups, there was no patient with serum anti-p53 antibody positive value that exceeded the standard value. In the pancreatic cancer group, the level in 27 patients was below the measurement sensitivity, and for the patients with a level that was highly sensitive, the mean value was 0.716 U/mL (0.41–1.23 U/mL). In the biliary tract cancer groups, the level was below the measurement sensitivity in 9 patients, while the mean value for patients with a level that was highly sensitive was 0.716 U/mL (0.41–1.20 U/mL). In the pancreatobiliary cancer group, the mean value for patients with a level that was highly sensitive was 0.716 U/mL (0.41–1.23 U/mL) (Tables 3, 4, 5, and 6).

### 4.3. p53 Immunohistochemistry

Rate of p53 protein overexpression in the 16 patients (surgical resection specimens from 5 patients and biopsy specimens from 11 patients) of the pancreatic cancer group that could be tested was 43.7% (7 patients) and in the 9 patients (surgical resection specimens from 2 patients and biopsy specimens from 7 patients) of the biliary tract cancer group was 55.5% (5 patients). In the pancreatobiliary cancer group, the rate was 48.0% (Tables 7 and 8). Among the patients with benign pancreatobiliary diseases (biopsy specimens from 9 patients), the rate of p53 protein overexpression was 0%.

## 5. Discussion

The p53 gene encodes a 53-kd DNA binding nuclear phosphoprotein with a short half-life that negatively regulates cell growth and proliferation, and its alteration or loss is thought to deprive cells of these inhibitory signals [22–24]. Several investigators have reported that pancreatic ductal cancers frequently show mutations of the p53 gene [25–27] as in biliary tract cancer [28–31]. Thus, there may be an obvious potential for the measurement of p53 gene products, namely, p53 protein, to diagnose pancreatobiliary malignancy.

So far, the main procedures for detecting p53 gene mutation are the analysis of gene sequences from RNA eluted from tissues such as resected materials and the detection of the mutant p53 protein by immunostaining. Induction of serum anti-p53 antibody against mutant p53 protein in cancer cells has been reported previously [32, 33]. Development of the ELISA kit that detects anti-p53 antibody is expected to be clinically useful as a screening test because it will enable the easy prediction of gene mutation.

According to the review by Soussi [34] in 2000, there is no report on biliary tract cancer. In terms of pancreatic cancer, previous reports showed various levels of positive rate of serum p53 antibodies, ranging from 4% to 27% [34]. In addition, Shimada et al. [16] investigated 1085 solid tumor patients with a total of 15 types of solid tumors in 2003 and reported a positive rate of 16% for biliary tract cancer and 10% for pancreatic cancer. With regard to the ratio of p53 mutations in pancreatobiliary cancers, several investigators have reported that they emerged in approximately 40% [25–31]. On the other hand, to date, there are many articles on the usefulness of p53 immunostaining for the diagnosis of pancreatic cancers [35–39] and biliary tract cancers [33–37]. The ratio of protein overexpression of pancreatobiliary cancers is approximately 60% though the range is from 40% to 80% [35–44]. There is discrepancy of positive rate between p53 mutation and protein expression. One of reason for this may be caused by the criteria for p53 overexpression. Current commercially available antibodies for P53 stains are both the wild-type and mutant p53 proteins. Nevertheless, p53 overexpression is thought to reflect p53 mutation. In fact, two reports have revealed that p53 protein overexpression correlates well with gene mutation in gallbladder cancer [45] and cholangiocarcinoma [46]. In the present study, we strictly defined that more than 70% of positive cells indicates overexpression, suggesting p53 mutation. As a result, the p53 overexpression positive rate was 43.7% in pancreatic cancer and 55.5% in biliary tract cancer. Surprisingly, in the present study, no anti-p53 antibody was detected in both pancreatic cancer and biliary tract cancer though the levels of CA19-9 or CEA were elevated in the same cases. We guessed that the reasons why p53 overexpression could not induce serum antibody were that p53 overexpression does not necessarily induce serum antibody and possibly some immunological malformation might be present in the patients with pancreatobiliary cancers.

Considering the clinical application of the current serum p53-antibody measurement and p53 immunostaining to diagnose pancreatobiliary malignancy, p53 immunostaining appears to be useful compared to serum anti-p53 antibody because of higher positive rate. In the present study, although p53 immunostaining was also used only for surgically resected specimens, the application of p53 immunostaining using biopsy specimens may be useful to distinguish benign from malignant as we previously described [44]. Therefore, we should compare serum anti-p53 antibody with p53 immunostaining using biopsy specimens as a next step in the near future.

There are some limitations in this study because of the small sample size, lack of a control group, and no analysis of p53 gene mutation.

In conclusion, our study clarified that serum anti-P53 antibody measurement does not contribute to the diagnosis of pancreatobiliary cancers. Instead, traditional p53 immunostaining still appears to be valuable in combination with standard procedures.

## Figures and Tables

**Table 1:** Clinical characteristics of patients with pancreatobiliary cancer.

	Pancreatic cancer (*N* = 35)	Biliary tract cancer (*N* = 12)
Age (Yr) Median (range)	69.3 (51–83)	72.3 (54–88)
Sex (no.) male/female	19/16	8/4
Stage (no.) (I/II/III/IVa/IVb)	2/0/4/13/16	0/1/2/3/6
Primary tumor site (no.)	Head 16 Body 12 Tail 7	Gallbladder 4 Bile duct 8

**Table 2:** Clinical characteristics of patients with benign disease.

*N *	31	
Age (Yr)	65.0	
Median (range)	(35–86)	
Sex (no.) male/female	25/6	
Disease (*n*)	CBD stone Chronic pancreatitis Bile duct stricture Autoimmune pancreatitis (AIP) Adenomyomatosis (ADM) of the gallbladder Pancreatic pseudocyst Pancreaticobiliary maljunction Pancreatic duct stricture	13 6 4 2 2 2 1 1

**Table 3:** Assay of p53 antibody, CEA, and CA19-9.

	Pancreatic cancer		Biliary tract cancer		Pancreatobiliary cancer	
	Mean ± SD^∗1^	CV^∗2^	Mean ± SD^∗1^	CV^∗2^	Mean ± SD^∗1^	CV^∗2^
p53 antibody (U/mL)	0.716 ± 0.30	0.42	0.716 ± 0.42	0.59	0.716 ± 0.31	0.44
CEA (ng/mL)	38.33 ± 146.8	3.83	21.42 ± 25.0	1.16	34.01 ± 127.05	3.73
CA19-9 (U/L)	3142.9 ± 9146.8	2.91	5859.8 ± 16446.5	2.79	3836.6 ± 11311.7	2.94

^∗1^SD: standard deviation.

^∗2^CV: coefficient of variation.

**Table 4:** Detection of p53 antibody, CEA, and CA19-9.

	Pancreatic cancer						
	Sensitivity	Specificity	PPV^∗1^	NPV^∗2^	FPR^∗3^	FNR^∗4^	Accuracy
p53 antibody	0.0%	83.8%	0.0%	42.6%	16.1%	100.0%	39.3%
CEA	42.8%	93.5%	88.2%	59.1%	6.4%	57.1%	66.6%
CA19-9	85.7%	90.3%	90.9%	84.8%	9.6%	10.6%	87.8%

^∗1^PPV: positive predictive value.

^∗2^NPV: negative predictive value.

^∗3^FPR: false positive rate.

^∗4^FNR: false negative rate.

**Table 5:** Detection of anti-p53 antibody, CEA, and CA19-9.

	Biliary tract cancer						
	Sensitivity	Specificity	PPV^∗1^	NPV^∗2^	FPR^∗3^	FNR^∗4^	Accuracy
p53 antibody	0.0%	83.8%	0.0%	42.6%	16.1%	100.0%	39.3%
CEA	41.6%	93.5%	29.4%	59.1%	6.4%	58.3%	51.5%
CA19-9	91.6%	93.5%	33.3%	84.8%	9.6%	8.3%	59.0%

^∗1^PPV: positive predictive value.

^∗2^NPV: negative predictive value.

^∗3^FPR: false positive rate.

^∗4^FNR: false negative rate.

**Table 6:** Detection of anti-p53 antibody, CEA, and CA19-9.

	Pancreatobiliary cancer						
	Sensitivity	Specificity	PPV^∗1^	NPV^∗2^	FPR^∗3^	FNR^∗4^	Accuracy
p53 antibody	0.0%	83.8%	0.0%	35.6%	16.1%	100.0%	33.3%
CEA	74.0%	93.5%	90.9%	90.9%	6.4%	57.4%	62.8%
CA19-9	87.2%	90.3%	93.1%	93.1%	9.6%	12.7%	88.4%

^∗1^PPV: positive predictive value.

^∗2^NPV: negative predictive value.

^∗3^FPR: false positive rate.

^∗4^FNR: false negative rate.

**Table 7:** Positive rate of serum p53 antibody and p53 overexpression.

	Pancreatic cancer	Biliary tract cancer	Pancreatobiliary cancer
p53 antibody	0/35 (0%)	0/12 (0%)	0/47 (0%)
p53 overexpression	7/16 (43.7%)	5/9 (55.5%)	12/25 (48.0%)

**Table 8:** Detection of p53 immunohistochemical analysis.

	Sensitivity	Specificity	PPV^∗1^	NPV^∗2^	FPR^∗3^	FNR^∗4^	Accuracy
Pancreatic cancer	43.7%	100.0%	0.0%	56.2%	100.0%	35.7%	57.1%
Biliary tract cancer	55.5%	100.0%	0.0%	44.4%	100.0%	50.0%	69.2%
Pancreatobiliary cancer	48.0%	100.0%	0.0%	52.0%	100.0%	40.9%	61.7%

^∗1^PPV: positive predictive value.

^∗2^NPV: negative predictive value.

^∗3^FPR: false positive rate.

^∗4^FNR: false negative rate.
